# Graph Theory Analysis Reveals Resting-State Compensatory Mechanisms in Healthy Aging and Prodromal Alzheimer’s Disease

**DOI:** 10.3389/fnagi.2020.576627

**Published:** 2020-10-22

**Authors:** Qumars Behfar, Stefan Kambiz Behfar, Boris von Reutern, Nils Richter, Julian Dronse, Ronja Fassbender, Gereon R. Fink, Oezguer A. Onur

**Affiliations:** ^1^Department of Neurology, Faculty of Medicine and University Hospital Cologne, University of Cologne, Cologne, Germany; ^2^Cognitive Neuroscience, Research Centre Jülich, Institute of Neuroscience and Medicine (INM-3), Jülich, Germany; ^3^Laboratory for Innovation Science at Harvard (LISH), Harvard University, Cambridge, MA, United States

**Keywords:** compensation, degree centrality, Brainnetome atlas, healthy aging, mild cognitive impairment

## Abstract

Several theories of cognitive compensation have been suggested to explain sustained cognitive abilities in healthy brain aging and early neurodegenerative processes. The growing number of studies investigating various aspects of task-based compensation in these conditions is contrasted by the shortage of data about resting-state compensatory mechanisms. Using our proposed criterion-based framework for compensation, we investigated 45 participants in three groups: (i) patients with mild cognitive impairment (MCI) and positive biomarkers indicative of Alzheimer’s disease (AD); (ii) cognitively normal young adults; (iii) cognitively normal older adults. To increase reliability, three sessions of resting-state functional magnetic resonance imaging for each participant were performed on different days (135 scans in total). To elucidate the dimensions and dynamics of resting-state compensatory mechanisms, we used graph theory analysis along with volumetric analysis. Graph theory analysis was applied based on the Brainnetome atlas, which provides a connectivity-based parcellation framework. Comprehensive neuropsychological examinations including the Rey Auditory Verbal Learning Test (RAVLT) and the Trail Making Test (TMT) were performed, to relate graph measures of compensatory nodes to cognition. To avoid false-positive findings, results were corrected for multiple comparisons. First, we observed an increase of degree centrality in cognition related brain regions of the middle frontal gyrus, precentral gyrus and superior parietal lobe despite local atrophy in MCI and healthy aging, indicating a resting-state connectivity increase with positive biomarkers. When relating the degree centrality measures to cognitive performance, we observed that greater connectivity led to better RAVLT and TMT scores in MCI and, hence, might constitute a compensatory mechanism. The detection and improved understanding of the compensatory dynamics in healthy aging and prodromal AD is mandatory for implementing and tailoring preventive interventions aiming at preserved overall cognitive functioning and delayed clinical onset of dementia.

## Introduction

Opposing effects of aging on brain functions have been reported: elderly individuals show decreased activity in some brain regions but increased activity in others (Cabeza and Dennis, 2012). These findings were challenging to the traditional assumption that aging is only linked with a simple pattern of cognitive and neural decline, supported by a body of research demonstrating an overall reduction of structural and functional brain integrity in Alzheimer’s Disease (AD).

A few studies investigated how the brain of AD patients reorganizes itself, which was interpreted as effects of brain plasticity (delEtoile and Adeli, 2017). Contrary to the initial belief, these and other studies have shown that “neuroplasticity” is not solely confined to children (Dennis et al., 2013) but is also observable in the healthy aging brain (Fuchs and Flügge, 2014) and even under the circumstances of neurodegeneration (Enciu et al., 2011), including Alzheimer’s Disease (delEtoile and Adeli, 2017). The latter findings gave rise to the concept of “neuronal compensation.”

Despite its popularity, the concept of compensation remains somewhat ambiguous, as the underlying neural mechanisms to date are still poorly understood. At least in part, this elusiveness is due to the complexity of defining the characteristics of compensation and the challenge to assess these characteristics *in vivo* (Gregory et al., 2017). To this end, various theoretical models of compensation in healthy aging and in the presence of neurodegeneration (Gregory et al., 2017) have been suggested. Most current theories of compensation were developed in task-based contexts, while compensatory processes in resting-state networks in healthy brain aging and early neurodegeneration have only rarely been addressed. However, resting-state studies offer several advantages over task-based ones, as they place low demands on the experimental design, compliance, instructions to be followed by participants, and training demands.

Cabeza et al. suggested that some essential criteria ought to be fulfilled for an observed increase in connectivity to reflect compensation (Cabeza et al., 2018). For example, increased connectivity should directly or indirectly be related to a neural resource deficiency or the supply demand gap (Lövdén et al., 2010; Cabeza and Dennis, 2012). The latter could be due to brain atrophy, reduced cerebral perfusion, or neurotransmitter deficiency (Cabeza et al., 2018). In the context of resting-state network connectivity, we propose four criteria to indicate compensatory mechanisms. First, the brain region must show a significant increase in functional connectivity. Second, the increase in functional connectivity must be accompanied by a decline of brain integrity in that region, e.g., volume reduction (Cabeza and Dennis, 2012). Third, the region must be specifically related to cognitive processing, to rule out non-selective neural recruitment (Cabeza, 2002; Logan et al., 2002). Finally, the connectivity increase of that region must be positively correlated with cognitive performance, thereby differentiating compensation from unspecific and maladaptive recruitment, in which greater connectivity is not associated with better cognitive performance (Cabeza and Dennis, 2012) or even with worse performance (Bakker et al., 2012).

We incorporated graph theory analysis in our novel criterion-oriented framework to investigate the resting-state compensation in healthy brain aging and prodromal AD. We hypothesized that during both healthy aging and MCI with biomarkers indicative of Alzheimer’s disease, brain regions show compensatory mechanisms, characterized by a significant increase of degree centrality, despite atrophy. Moreover, we assumed that degree centrality increases in these regions would be positively correlated with cognitive performance, indicating effective compensation.

## Materials and Methods

### Participants

The current study was part of the RIMCAD-study (*Retroactive Interference during Memory Consolidation in Aging and Dementia*) conducted by the Memory Clinic Köln Jülich. The Ethics Committee of the Faculty of Medicine of Cologne University had approved the RIMCAD-study. Out of the RIMCAD study’s larger pool of participants, three experimental groups were defined for the present study: (i) Fifteen young healthy controls (young HC), (ii) fifteen senior healthy controls (senior HC), and fifteen MCI patients (see Table 1). As MCI encompasses a heterogeneous population (Petersen et al., 2001), we solely recruited prodromal AD participants per the IWG-2 criteria and Dubois et al. (2014, 2016), with at least one positive AD biomarker. Biomarkers suggestive of AD included an abnormal amyloid deposition (either assessed by positron-emission-tomography or by an abnormal concentration of cerebrospinal fluid (CSF) β-amyloid 42), or an abnormal concentration of phospho-tau or a total tau/β-amyloid 42 ratio greater than 0.52 in CSF samples (Duits et al., 2015). All of the MCI patients were amnestic and 40% of them showed additional deficits in executive functions. Informed written consent had been obtained by all participants and upon completion of the study, they received financial compensation. All participants were right-handed, which was assessed using the Edinburgh Handedness Inventory (Oldfield, 1971) and had a normal or corrected-to-normal vision. As for the exclusion criteria, the participants were screened for neurological and psychiatric disorders, including a history of traumatic brain injury, epilepsy, Parkinson’s Disease, Multiple Sclerosis, depression, mania, or schizophrenia. Besides, past and present drug or alcohol abuse, as well as respiratory, cardiovascular, and gastro-intestinal, or kidney-related diseases were exclusion criteria. Moreover, contraindications to undergoing MRI, such as claustrophobia, non-removable piercings, a pacemaker, or magnetic implants, were checked beforehand. Notably, we ascertained our sample size by performing a *post-hoc* analysis using G^∗^Power 3.1 (Faul et al., 2007) and IBM SPSS, version 23.0.

**TABLE 1:** Demographic data of MCI, senior HC, and young groups.

	Young HC	Senior HC	MCI
sample size (n)	15	15	15
Sex (%male)	60	60	60
Age	24.4 ± 2.85	67.26 ± 8.11	71.13 ± 5.76
Education (years)	15.53 ± 4.44	14.6 ± 3.62	12.46 ± 3.65
MMSE	N/A	29.53 ± 0.61	25.14 ± 3.18

### MRI Data Acquisition and Preprocessing

All participants in the study underwent MRI imaging at the Research Centre Jülich. Structural MRI and resting-state functional MRI were collected at a 3T MAGNETOM Trio scanner (Siemens, Erlangen, Germany). T1-weighted structural images were obtained using a rapid gradient echo sequence with the following parameters: repetition time (TR) = 2250 ms, echo time (TE) = 3.03 ms, flip angle (FA) = 9°, field of view (FOV) = 256 × 256 mm ^2^, voxel size = 1 mm isotropic, 176 gapless interleaved sagittal slices. During the resting-stage image acquisition, which took 7 min, patients were instructed to stay awake with open eyes and not to think of anything particular. For the functional images echo planar imaging (EPI) with the following parameters was used: TR = 3000 ms, TE = 30 ms, FA = 90°, FOV = 200 × 200, voxel size = 2.5 × 2.5 × 2.8, interleaved oblique slices parallel to the infra-supratentorial line with a gap of 0.28 mm.

Data were preprocessed using the default preprocessing pipeline of the CONN toolbox (Whitfield-Gabrieli and Nieto-Castanon, 2012). The first four images of 155 volumes were removed to allow the signal to reach equilibrium. Functional images were realigned to the first acquired volume in the session. Subsequently, echo planar images (EPIs) were co-registered to the high-resolution T1 structural image, and normalized to the Montreal Neurological Institute (MNI) stereotactic space and resampled at 2 × 2 × 2 mm^3^ voxel size. The normalized images were spatially smoothed with an 8-mm full-width at half maximum (FWHM) isotropic Gaussian kernel. Head movement parameters were checked individually and excluded at a relative displacement criterion of ± 3 mm. After preprocessing the MRI data, 246 ROI were extracted using the Brainnetome Atlas (Fan et al., 2016) and imported into the CONN toolbox.

To address motion-related artifacts (Conwell et al., 2018), we incorporated frame-wise displacement (FD) calculated proposed by Jenkinson et al. (2002) as a covariate of no interest in our models. The method by Jenkinson et al. is preferable over other methods of FD calculation due to its consideration of voxel-wise differences in its derivation (Yan et al., 2013).

### Graph Theory Analysis

**FIGURE 1:**
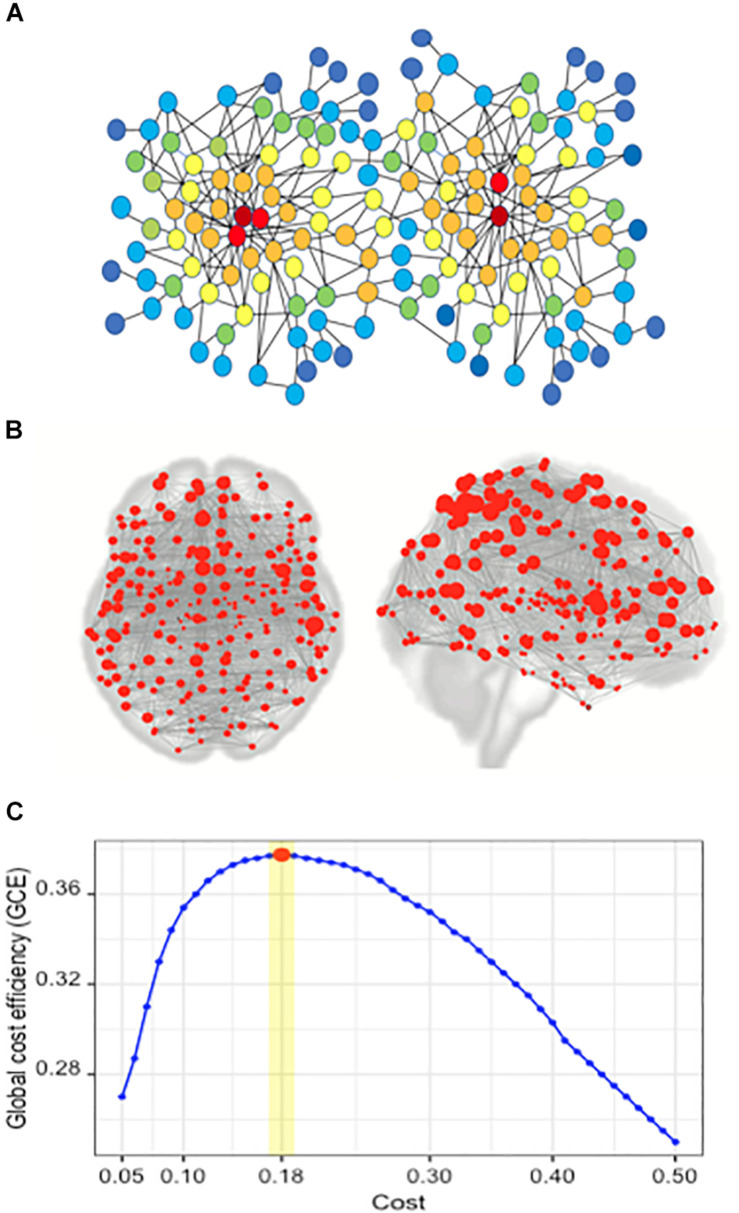
Network and the concept of degree centrality; the brain network of the Brainnetome atlas’ ROIs; finding the optimal cost. **(A)** In the illustrated network, the red nodes have the highest degree centrality, and the color spectrum from red to blue represents the gradual reduction of degree centrality. **(B)** Red circles represent the nodes in a brain network, composed of 246 nodes. In this network, each node is one of the 246 ROIs of the Brainnetome Atlas. **(C)** Plot of global cost efficiency vs. Cost averaged across all subjects in young HC, senior and MCI groups.

Graph theory is a standard framework for the mathematical representation of networks. A network can be represented as a graph by G (N, K), with N, indicating the number of nodes and K as the number of edges in the graph G. The degree centrality (DC) is a simple measurement of connectivity between a single node and all other nodes in a network, representing the importance of a node in a network. The degree of node *i* is the number of edges connected to it and is calculated *as k_*i*_* = Σ*jεGa_*ij*_* (*a*_*ij*_ is the *i*th row and *j*th column element of adjacency matrix *A*) (Wang, 2010). In graph theory and network analysis, indicators of centrality identify the major nodes within a graph and a hub is a node with a number of edges that exceeds the average (see Figure 1).

In order to apply graph theory analysis to fMRI scans, BOLD (blood oxygen level dependent) time series of brain activity were used, and the 246 imported Brainnetome Atlas ROIs served as the nodes of the network.

We incorporated Brainnetome Atlas (Fan et al., 2016) on CONN toolbox (Whitfield-Gabrieli and Nieto-Castanon, 2012) to generate connectivity matrices, by averaging the time series of the BOLD (blood oxygenation level dependent) signals of all voxels in each ROI and calculating the Pearson’s correlation of these average signals between ROIs.

The proportion of the remained robust connections to the total number of connections is defined as Cost, ranging from 0 to 1. On one hand, setting very large values to Cost results in keeping weaker edges and noisy connections and on the other hand, assigning very small values to Cost removes too many edges, which consequently generates a disconnected graph. By adjusting the Cost as a threshold in a network of *n* nodes (*N*), one can optimize the global cost efficiency (GCE) of the network (Bassett et al., 2009; Bullmore and Sporns, 2009; Khazaee et al., 2016), which is calculated as:

(1) G⁢C⁢E=G⁢E-C⁢o⁢s⁢t

(2) $$G\,E=\frac{1}{n}\,\sum_{i\in N}E_{i}=\frac{1}{n}\,\sum_{i\in N}\frac{\sum_{j\in N,j\neq i}d_{i\,j}^{-1}}{n-1}$$

Where G*E* and *E*_*i*_ are, respectively, the global efficiency and the efficiency of node *i*. And, *d*_*ij*_ is the shortest path length between nodes *i* and *j*.

Furthermore, the weighted connectivity matrices can be transformed into binary ones by applying an optimal threshold on connectivity matrices (Bassett et al., 2009; Dimitriadis et al., 2010).

The CONN toolbox allows the computation of both global and nodal graph measures on binary and weighted networks. At the single-subject level, we performed a ROI-to-ROI analysis incorporating all ROIs of the Brainnetome Atlas (Fan et al., 2016). We explored the optimal Cost value which maximizes the GCE. Different Cost values ranging from 0.05 to 0.5 by a step of 0.01 were examined. The optimal values of Cost were 0.18 ± 0.01 across all subjects; a maximum GCE of 0.37 was achieved (Figure 1). Then, the generated weighted connectivity matrices were transformed into binary matrices using a Cost of 0.18, as the mean of the optimal range on positive correlations.

Following ROI-to-ROI analyses at the subject level, we conducted analyses at the group level. Graph theory analyses on the three groups of young HC, senior HC, and MCI subjects were performed, and the adjacency matrices and network measures of each ROI were exported. Between-group differences on degree centrality were determined using two-tailed *t*-tests with a *p* < 0.05 (FDR-corrected) in two separate contrasts including (1) young HC vs. senior HC and MCI, (2) senior HC vs. MCI. For the first contrast, sex and education were applied as covariates of no interest, for the second contrast age was included in addition to sex and education. After group level comparisons, the DC measure of the ROIs with significantly higher DC was extracted for further correlation analysis with neuropsychological tests.

### Brainnetome Atlas

The Brainnetome Atlas (Fan et al., 2016) includes 246 ROIs (210 cortical and 36 subcortical subregions) (see Figure 1), which are assigned to brain functions based on meta-analyses of tasked-based functional imaging techniques (Fan et al., 2016). Most human brain atlases lack fine-grained parcellations and fail to provide all aspects of functional connectivity. Using non-invasive multimodal imaging techniques, the Brainnetome Atlas was designed to provide a connectivity-based parcellation framework, which identifies the subdivisions of the human brain, revealing new dimensions of connectivity architecture. In particular, the atlas combines brain connectivity with cytoarchitecture and other microscale information. The delineated structures in the Brainnetome Atlas are mapped to mental processes by referring to the BrainMap database (Laird et al., 2009; Balsters et al., 2014; Fox et al., 2014), to provide an initial estimate of the mental processes sustained by each cortical and subcortical region of the Brainnetome Atlas (Fan et al., 2016). The functional characteristics of each subarea in the Brainnetome Atlas are based on the behavioral domains and paradigm class meta data labels of the BrainMap database^1^, employing forward and reverse inferences (Eickhoff et al., 2011; Cieslik et al., 2013; Clos et al., 2013; Fox et al., 2014; Fan et al., 2016).

### Volumetric Analysis

To locate the regions fulfilling criteria for compensation, as defined in the introduction, and to distinguish ROIs with significant atrophy, a volumetric analysis was performed using the computational anatomy toolbox (CAT12, Version 12.1)^2^, an extension of statistical parametric mapping (SPM12; Wellcome Centre for Human Neuroimaging)^3^ implemented in MATLAB R2015b (The MathWorks, Natick, United States). After segmentation of all T1 images into gray matter (GM), white matter (WM), and cerebrospinal fluid, all images were normalized to the MNI space using DARTEL with six iterations and the integrated DARTEL template in MNI space (Mechelli et al., 2005; Ashburner, 2009) using the registration step, local GM and WM volumes were preserved by modulating their Jacobian determinants. Subsequently, the normalized GM were smoothed by Gaussian filter (FWHM = 8 × 8 × 8 mm), from which the GM volumes of the ROIs with a significant increase of DC as generated in the group contrast were extracted for the statistical analysis.

### Neuropsychological Tests

Out of a larger pool of neuropsychological tests within the RIMCAD-study (for a comprehensive description see Conwell et al., 2018), we selected the Verbal Learning and Memory Test (VLMT), a German version of the Rey Auditory Verbal Learning Test (RAVLT) (Lux et al., 1999) as a test of memory performance (Zhao et al., 2015), and The Trail Making Test (TMT) (Rodewald et al., 2012) as an indicator of cognitive flexibility (Kinsella et al., 2007).

We aimed to refine and uniform the test results by generating a total VLMT value by averaging the standardized z-scores of the VLMT trials I–V (total learning), VI (recall after interference), and VII (delayed recall), and a delta TMT value by subtracting the TMT-B from the TMT-A values.

### Correlation Analysis Between Compensatory ROIs and Neuropsychological Tests

Per correlation analysis, we examined if a higher connectivity was correlated with a better performance, as postulated in the fourth criterion in the introduction. To assess the correlation, we first exported all the graph measures following the graph theory analysis embedded in the group level result section of the CONN toolbox. After that, we specifically extracted the DC measures of all the significant ROIs in the senior HC and MCI subjects. Next, we tested the correlation between DC of compensatory ROIs and the neuropsychological test results in senior HC and MCI subjects using a linear regression function with least square fit, implemented in MATLAB R2015b (The MathWorks, Natick, United States), and corrected for multiple comparisons. For visualization purposes, R (R Core Team, 2013) along with ggplot2 package (Wickham, 2016) has been used.

### Seed-to-ROI Analysis

We also performed Seed-to-ROI analyses using the compensatory ROIs as seeds and all 246 ROIs in the Brainnetome Atlas as target ROIs. Seed-to-ROI correlation matrices were defined as the Fisher-transformed bivariate correlation coefficients between two ROIs BOLD signals^4^.

$$r\,\left(i,j\right)=\frac{\sum_{t}R_{i}\,\left(t\right)\,R_{j}\,\left(t\right)}{\sqrt{\sum_{t}R_{i}\,{\left(t\right)}^{2}\,\sum_{t}R_{j}\,{\left(t\right)}^{2}}}$$

$$Z\,\left(i,j\right)=t\,a\,n\,h^{-1}\,r\,\left(i,j\right)$$

*Ri(t)* = *BOLD signals within i^*th*^ ROI, centered to zero mean*.*r(i,j)* = *correlation coefficients between i^*th*^ and j^*th*^ ROIs*.*Z(i,j)* = *Fisher-transformed correlation coefficient*.

Seed-to-ROI correlation analyses were conducted at the single-subject level of the CONN toolbox as bivariate correlations without weighting.

## Results

We assessed the imaging and neuropsychological data of all participants (*N* = 45) in three groups of young HC, senior HC and MCI. All three groups did not differ significantly in education level and sex. There was no significant difference in age among the senior HC and MCI groups (*p* > 0.05). In each step of the following analyses the normal distribution of the data was approved by the Shapiro-Wilk test in R.

### Graph Theory Analysis and the ROIs With a Significant Increase of DC

**FIGURE 2:**
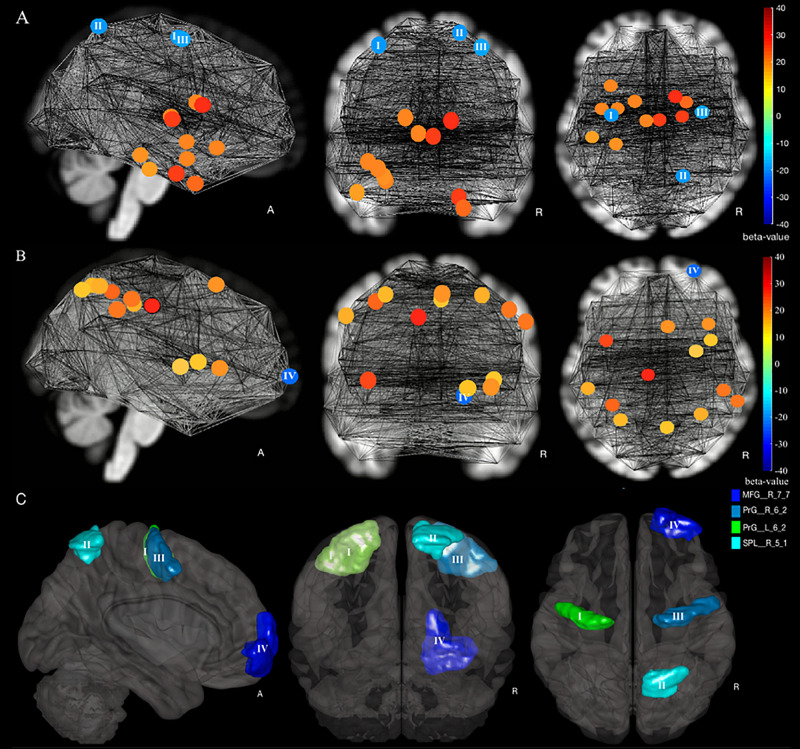
Increase and decrease of degree centrality in a comparison of young HC, senior HC, and MCI. **(A)** Young HC vs. senior HC and MCI contrast, in which blue shade circles represent a significant increase, and red shade circles represent a significant decrease of DC in Senior HC and MCI subjects compared to young HC (*p* < 0.05, FDR-corrected). **(B)** Senior HC vs. MCI contrast, in which blue shade circles represent a significant increase, and red shade circles represent a significant decrease of DC in MCI subjects compared to senior HC (*p* < 0.05, FDR-corrected). The significant ROIs are according to labeling by the Brainnetome atlas (I) PrG_L_6_2, (II) SPL_R_5_1, (III) PrG_R_6_2, and (IV) MFG_R_7_7. **(C)** All four compensatory ROIs stem from the two intergroup contrasts.

**FIGURE 3:**
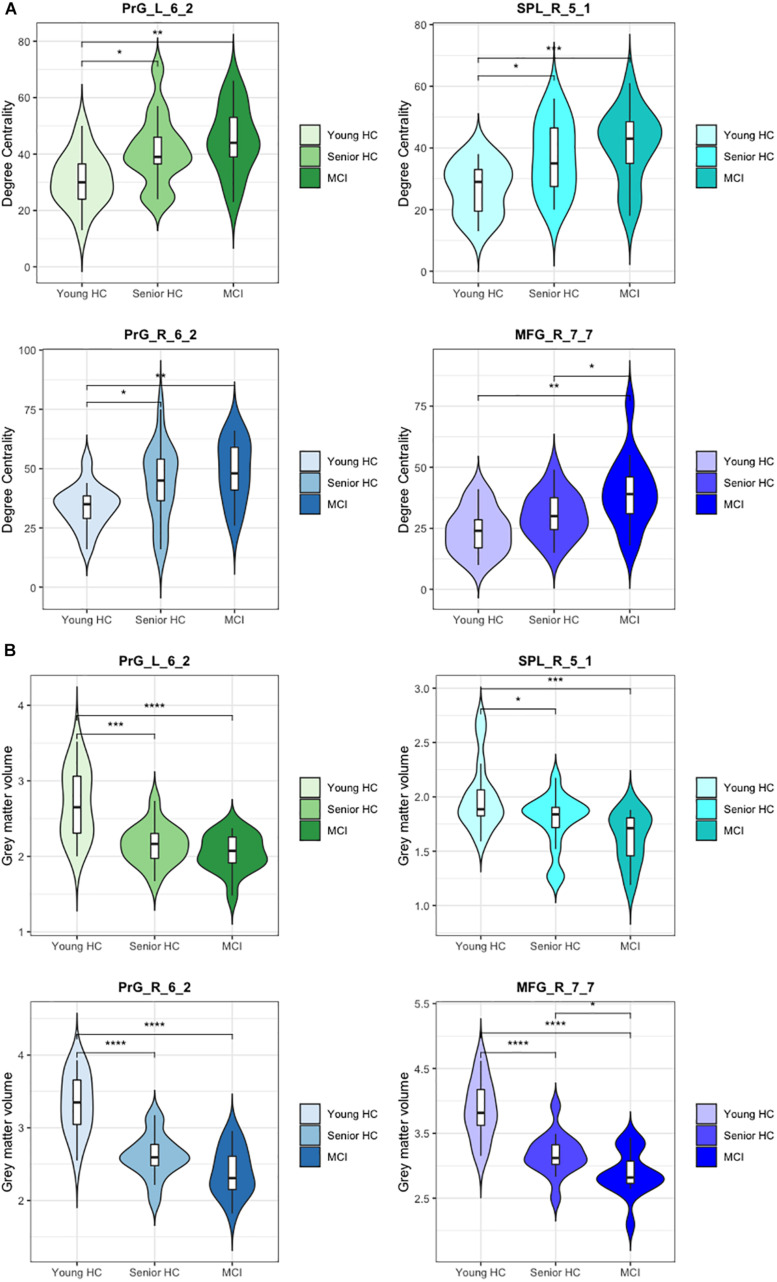
Degree centrality and gray matter volume of compensatory ROIs. Illustration of **(A)** the degree centrality and **(B)** the gray matter volume of the right and the left precentral gyri caudal dorsolateral area 6 (Brainnetome label: PrG_R_6_2 and PrG_L_6_2), the superior parietal lobe, rostral area 7 (Brainnetome label: SPL_R_5_1), and the right middle frontal gyrus, lateral area 10 (Brainnetome label: MFG_R_7_7). (*) indicates statistical significance (*p* < 0.05, FDR-Corrected), (**) indicates statistical significance (*p* < 0.01, FDR-Corrected), (***) indicates statistical significance (*p* < 0.005, FDR-Corrected), (****) indicates statistical significance (*p* < 0.001, FDR-Corrected).

We observed an increase of DC in the senior HC and MCI group vs. young HC in three ROIs: the right superior parietal lobule, rostral area 7 (Brainnetome label: SPL_R_5_1), the right and left precentral gyri caudal dorsolateral area 6 (Brainnetome label: PrG_R_6_2 and PrG_L_6_2) (Figures 2A,C, 3A, increases of DC depicted in circles of blue shades, decreases in red shades; see also Table 2). Furthermore, the comparison between the senior HC and the MCI group revealed an increase of DC in the right middle frontal gyrus, lateral areal 10 (Brainnetome label: MFG_R_7_7) (Figures 2B,C, 3A and Table 2).

**TABLE 2:** The coordinates of the ROIs with a significant increase of DC in the intergroup contrasts.

Contrast	Brainnetome atlas label	Region	Behavioral domain according to the Brainnetome atlas
Senior HC + MCI > young HC (*p* < 0.05, FDR-corrected)	PrG_L_6_2	Left precentral gyrus, caudal dorsolateral area 6	Spatial cognition, action execution
Senior HC + MCI > young HC (*p* < 0.05, FDR-corrected)	SPL_R_5_1	Right superior parietal gyrus, rostral area 7	Working memory, somatic and spatial cognition, attention, action execution
Senior HC + MCI > young HC (*p* < 0.05, FDR-corrected)	PrG_R_6_2	Right precentral gyrus, caudal dorsolateral area 6	Somatic and spatial cognition, action execution
MCI > senior HC (*p* < 0.05, FDR-corrected)	MFG_R_7_7	Right middle frontal gyrus, lateral area 10	Cognition, explicit memory
			

Importantly, the *post-hoc* estimation of our sample size using G^∗^Power 3.1 and IBM SPSS for α = 0.05 and the effect size = 0.39, which was calculated by MANOVA [*F*(4,40) = 6.5, *p* = 0.0001, partial η*^2^* = 0.39] from the results of the graph theory analysis, showed a power (1-ß) of 0.80.

### Volumetric Analysis of the Compensatory ROIs

As defined in our criteria of compensatory mechanisms, the respective ROIs must show a concurrent increase of DC with a decrease of gray matter volume. Having corrected for the total intracranial volume, volumetric analysis revealed a significant decrease in gray matter volume of the compensatory ROIs. As presented in Figure 3, all four compensatory ROIs (Table 2) exhibited significant volume reduction in the senior HC and MCI group in comparison to the young HC (*p* < 0.05, FDR-corrected). Although further gradual volume reductions were detected in the MCI group compared to the senior HC in all four compensatory ROIs, this was only significant in the right middle frontal gyrus (*p* < 0.05, FDR-corrected).

### Correlation Analysis Between DC, Total VLMT, and Delta TMT Values

**FIGURE 4:**
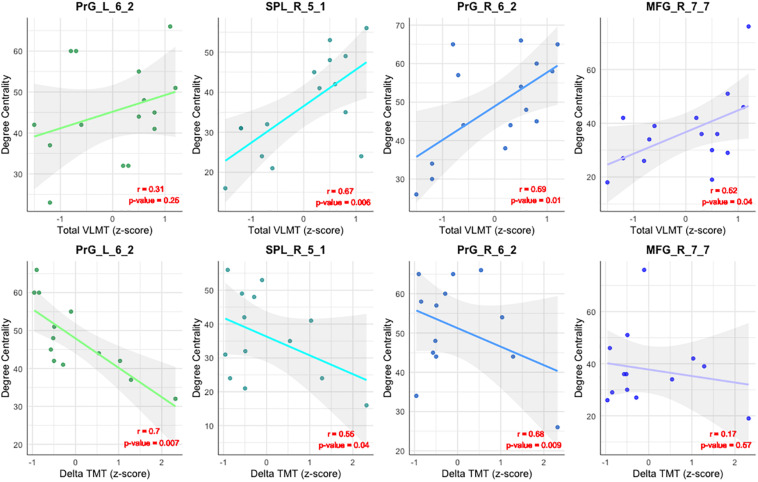
Correlation between DC and neuropsychological tests. Illustration of the correlation between the DC of the compensatory ROIs [the right and the left precentral gyri, caudal dorsolateral area 6 (Brainnetome label: PrG_R_6_2 and PrG_L_6_2), the superior parietal lobe, rostral area 7 (SPL_R_5_1), and the right middle frontal gyrus, lateral area 10 (Brainnetome label: MFG_R_7_7)], and the total VLMT score (upper row) and Delta TMT in MCI (lower row).

As shown in Table 3 and Figure 4, the DC of the right precentral gyrus, caudal dorsolateral area 6 (Brainnetome label: PrG_R_6_2), the right superior parietal lobe, rostral area 7 (Brainnetome label: SPL_R_5_1), and the right middle frontal gyrus, lateral area 10 (Brainnetome label: MFG_R_7_7) was significantly correlated with the total VLMT values in MCI group (*p* < 0.05, corrected for multiple comparisons). Likewise, the DC of the right and the left precentral gyri, caudal dorsolateral area 6 (Brainnetome label: PrG_R_6_2, PrG_L_6_2), and the right superior parietal lobe, rostral area 7 (Brainnetome label: SPL_R_5_1) was significantly correlated with the delta TMT values in MCI group (*p* < 0.05, corrected for multiple comparisons). As two participants in the MCI group failed to complete TMT assessment, two data points are missing. After correcting for multiple comparisons, the correlation between the DC measures of the compensatory ROIs, total VLMT, and delta TMT values revealed no significant association in senior HC (see Table 4).

**TABLE 3:** Correlation between DC of cognition-related compensatory ROIs, total VLMT, and delta TMT scores in MCI.

	Total VLMT		Delta TMT		Behavioral domain according to the Brainnetome atlas
ROI	*r*	*p-*value	*r*	*p-*value	
PrG_L_6_2	0.312	0.258	0.701	**0.007**	Spatial cognition, action execution
PrG_R_6_2	0.599	**0.018**	0.689	**0.009**	Somatic and spatial cognition, action execution
SPL_R_5_1	0.670	**0.006**	0.555	**0.048**	Working memory, somatic and spatial cognition, attention
MFG_R_7_7	0.522	**0.046**	0.173	0.571	Explicit memory

**Correlation with significant p-values are highlighted in bold. All p-values are corrected for multiple comparisons.**

**TABLE 4:** Correlation between DC of cognition-related compensatory ROIs, total VLMT, and delta TMT scores in senior HC.

	Total VLMT		Delta TMT		Behavioral domain according to the Brainnetome atlas
ROI	*r*	*p-*value	*r*	*p-*value	
PrG_L_6_2	0.286	0.301	0.368	0.178	Spatial cognition, action execution
PrG_R_6_2	0.260	0.350	0.464	0.082	Somatic and spatial cognition, action execution
SPL_R_5_1	0.274	0.322	0.002	0.995	Working memory, somatic and spatial cognition, attention
MFG_R_7_7	0.396	0.144	0.002	0.995	Explicit memory

**All p-values are corrected for multiple comparisons.**

### Seed-to-ROI Analysis

**FIGURE 5:**
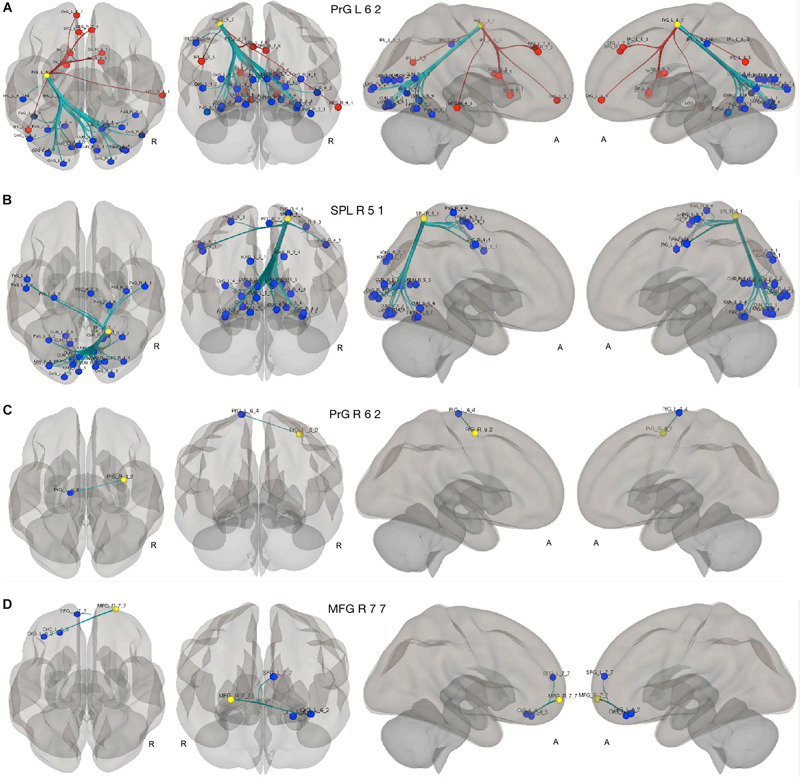
Seed to ROI analysis of the compensatory ROIs. The yellow circles represent the compensatory ROIs. The colored circles depict the ROIs with a significant increase (blue) or decrease (red) of connectivity with the corresponding compensatory ROIs in our two contrasts. **(A–C)** young HC > senior HC and MCI (*p* < 0.05, FDR-corrected). **(D)** Senior HC > MCI (*p* < 0.05, FDR-corrected).

As shown in Figure 5, the compensatory ROIs in the right and the left precentral gyri (Brainnetome label: PrG_R_6_2 and PrG_L_6_2) and the right superior parietal lobe (Brainnetome label: SPL_R_5_1) demonstrated a significant increase of connectivity with cognition-associated regions, including multiple ROIs in the occipital lobes, the cuneus, fusiform gyri, and pre- and postcentral gyri. The compensatory ROI in the right middle frontal gyrus (Brainnetome label: MFG_R_7_7) also showed a significant increase in connectivity with cognition-associated ROIs in the left superior frontal gyrus and the left orbital gyrus.

## Discussion

This study contributes to the ongoing discussion on compensatory mechanisms in neurodegenerative diseases. The primary purpose of this work was to draw attention to the dynamics and dimensions of compensation in healthy brain aging and MCI with AD biomarkers from a new perspective, namely the contribution of resting-state connectivity derived by graph theory. To the best of our knowledge, graph theory analysis has sparsely been used for the detection of compensatory effects in the AD continuum. We identified four cognition-related ROIs with characteristics of compensation in a resting-state-fMRI design, applying connectivity measures. The compensatory ROIs were the right superior parietal gyrus (rostral area 7), the right- and the left precentral gyri (caudal dorsolateral area 6), and the right middle frontal gyrus (lateral area 10). In these ROIs, we observed an increase of DC, indicating more robust connectivity, despite regional atrophy. This finding indicates that regional structural deterioration of the brain does not necessarily reflect regional brain function. Although the DC of the compensatory ROIs was well correlated with the cognitive performance in MCI patients, this correlation could not be observed in senior healthy controls. And, this finding is in good accordance with the concept of compensation, which is considered to reflect the brain’s attempt to compensate for the cognitive decline by increasing neural activity or connectivity. In the additional seed-to-ROI analyses using the aforementioned compensatory ROIs as seeds or hubs, we observed a significant increase of connectivity between these ROIs and cognition- and memory-associated ROIs in the caudal cuneus, the fusiform gyrus, the occipital polar cortex, the middle occipital gyrus, and the pre- and the postcentral gyri. The cognitive domains covered by these ROIs include language, semantic, and spatial cognition (Fan et al., 2016). Besides, and of particular relevance in the context of dementia, some of these ROIs were associated with learning and sequence recall (Fan et al., 2016), which is in line with our observation that the DC of the compensatory ROIs showed a significant correlation with memory, cognitive flexibility, and executive functioning, as derived by VLMT and TMT values.

In the context of task-based fMRI-experiments, various compensatory patterns of activity increases associated with brain aging have been proposed. According to one account, the hemispheric asymmetry of the prefrontal lobes observed in younger subjects is reduced in older adults. This pattern is called “Hemispheric Asymmetry Reduction in Older Adults” or HAROLD (Cabeza, 2002). Subsequent work suggested that such a mechanism may also be observed in the parietal lobe (Piefke et al., 2012). Another account suggested an increase of activity in prefrontal cortex in elderly individuals together with a. reduction of occipital lobe activity. This pattern was termed “Posterior-Anterior Shift with Aging” or PASA (Davis et al., 2008). Furthermore, a compensatory mechanism in healthy brain aging and AD was suggested by an increase in functional connectivity in the prefrontal cortex (Gregory et al., 2017) even though postmortem, *in vivo*, and brain imaging studies provided evidence for atrophy in the prefrontal cortex. This led to the frontal lobe hypothesis, which posits that cognitive inefficiencies in aging are predominantly due to the structural and functional deterioration of the frontal lobes (Cabeza and Dennis, 2012). The CRUNCH model (compensatory-related utilization of neural circuits) extends the models mentioned above and explains in general terms aging-related changes in activity related to compensation without restricting it to cerebral areas (Reuter-Lorenz and Cappell, 2008).

While the models mentioned above were revealed by task-based-fMRI designs and analyses focusing on regional activation rather than connectivity, our results based on connectivity showed comparable patterns. First of all, we were able to demonstrate successful compensation in the right middle frontal gyrus (lateral area 10) in MCI patients. Besides, we observed that in the transition from healthy aging to MCI, among all of the compensatory ROIs, volume reduction is only significantly pronounced in this prefrontal ROI, which vicariously addresses the frontal lobe hypothesis. We also demonstrated successful compensation in the right- and the left precentral gyri (caudal dorsolateral area 6), which have been consistently indicated in numerous studies as a key structure utilizing working memory tasks (Howard et al., 2003; Narayanan et al., 2005; Meltzer et al., 2008; Kirschen et al., 2010; Huang et al., 2013; Noy et al., 2015; Kambara et al., 2017). In line with these findings, there is some evidence, that an increase of resting-state nodal centrality in the right middle frontal gyrus and the right precentral gyrus in MCI might effectively serve as a compensatory mechanism, playing an essential role in MCI patients to recruit additional cognitive resources to achieve a normal level of cognition (Yao et al., 2010). Also, previous studies have provided evidence for task-specific compensatory recruitment of parietal lobe in brain aging (Huang et al., 2012; Piefke et al., 2012), and in this study, we could detect a resting-state compensatory recruitment of the right superior parietal gyrus (rostral area 7) in MCI patients. As an extension of the above-noted links between our results and previous studies, it is worth noting that our findings regarding compensatory connectivity change in the frontal and the parietal lobes could be considered as the resting-state counterparts of the HAROLD (Cabeza, 2002; Piefke et al., 2012) and/or CRUNCH (Reuter-Lorenz and Cappell, 2008)model of task-based compensation. By investigating the PASA phenomenon in the task-based and resting-state networks using graph measures, McCarthy et al. reported a bilateral increase of DC in the pre- and post-central gyri, and the superior parietal gyrus in the healthy aging and early AD (McCarthy et al., 2014). These findings are in line with our observations in the right- and the left precentral gyri (caudal dorsolateral area) and the right superior parietal gyrus (rostral area 7). McCarthy et al. (2014) have also observed a clear pattern of declining DC in posterior regions in the aged group, when compared to the young group. The latter lends support to our findings in the seed-to-ROI analysis, in which we observed an anterior-to-posterior tendency in the left precentral gyrus (caudal dorsolateral area 6) and the right superior parietal gyrus (rostral area 7).

Despite the converging evidence of our results and previous studies, it is worth noting that our findings regarding compensatory connectivity change in the frontal and parietal lobes could be considered as the resting-state counterparts of the HAROLD (Cabeza, 2002; Piefke et al., 2012) and/or CRUNCH (Reuter-Lorenz and Cappell, 2008) model of task-based compensation. Investigating the PASA phenomenon in task-based and resting-state networks using graph measures, McCarthy et al. reported a bilateral increase of DC in the pre- and post-central gyri, and the superior parietal gyrus in healthy aging and early AD (McCarthy et al., 2014). This is in line with our findings in the right- and the left precentral gyri (caudal dorsolateral area 6) and the right superior parietal gyrus (rostral area 7). McCathy et al. also reported a declining DC in posterior regions in the aged group when compared to a group of young subjects (McCarthy et al., 2014). These findings support our graph theory-based method, which reveals resting-state compensatory mechanisms in healthy aging and prodromal Alzheimer’s disease.

Finally, a critical issue for the relevance of these findings is their applicability and translation to the clinical and interventional settings. In general terms, non-invasive stimulation methods such as transcranial magnetic stimulation (TMS) may improve the neural performance of various brain regions (Solé-Padullés et al., 2006; Cotelli et al., 2008) and the compensatory ROIs might be used as targets of non-invasive stimulations.

There are several limitations to consider in our current study: First, our dataset is composed of only a small number of MCI patients characterized by beta-amyloid (Ab) and tau biomarkers. Thus, a study with a larger patient cohort is warranted to confirm these results. Second, we referred to the previous version of the A/T/N classification framework (Jack et al., 2016) as the diagnostic criteria for our participants. Whereas in the previous version of the framework (Jack et al., 2016), an isolated positive beta-Amyloid (A+) biomarker status was sufficient for AD classification, in the latest version (Jack et al., 2018), having both a beta-Amyloid (A+) and phospho-Tau (T+) biomarker status is required for an AD classification. Third, the CSF biomarkers were not available for the participants in the senior HC group. As some of the healthy elderly individuals might already have AD pathologies, including their biomarkers status would have been advantageous in interpreting the results. Fourth, in this study we only focused on group level comparisons. However, testing these criterions in an individual level have concrete preferences in terms of personalized medicine for the application in clinical practice, and it is an issue which merits further discussion. Group comparison fMRI studies have been extensively used to realize the generic aspect of brain function, usually by averaging across individuals to optimize the signal-to-noise ratio (SNR). Averaging the data has also a statistical advantage which is leveraged in group comparisons. However, the group comparison studies have fallen short of an appropriate characterization of brain function in the individual level. By far, the most frequently used approach to interpret the fMRI-derived results in an individual level is to relate them to other individual measures in the same subjects such as test scores or behavioral measures (Dubois and Adolphs, 2016; Dubois et al., 2016), which has also been the method of choice in our study. Nevertheless, another proposed approach is to shift from correlation analysis to a predictive (machine-learning inspired) framework to improve generalizability and interpretability of fMRI-derived results at the individual level (Linden, 2012; Gabrieli et al., 2015; Yarkoni and Westfall, 2017). Fifth, in this paper we exclusively investigated the applicability of our proposed framework of compensation using Brainnetome atlas in cerebral regions. However, the role of cerebellum in cognition, memory and learning (Schmahmann, 2010) could potentially expand the scope of cognitive compensation beyond the purview of cerebrum. Therefore, it will be essential to explore compensatory mechanism in cerebellum using other functional brain atlases with cerebellum coverage.

In conclusion, using combined graph theory analysis of resting-state fMRI data and volumetric analyses of structural MRI, we here show new characteristics of compensation in healthy brain aging and early neurodegeneration. Moreover, using an ROI-based atlas with fine parcellation, a more precise map of compensatory regions could be identified. The identified compensatory regions were well associated with the cognitive performance scores in MCI subjects, which offers new insights into the compensatory mechanism of memory and executive functions. Based on these findings, preferably more longitudinal studies with a broader spectrum of various categories and stages of cognitive impairment such as subjective memory impairment, early MCI, late MCI, amnestic vs. non-amnestic type of MCI, mild to moderate and severe AD are warranted to elucidate further the dynamics and dimensions of resting-state compensatory mechanism in neurodegenerative processes with cognitive decline. For future studies, analyses on individual level using a predictive machine-learning based framework seem to be a promising approach to further our understanding of the compensatory mechanisms.

## Data Availability Statement

The datasets for this article are not publicly available because public access to the dataset has not been yet approved. Requests to access the datasets should be directed to QB, qumars.behfar@uk-koeln.de.

## Ethics Statement

The studies involving human participants were reviewed and approved by the Ethics Committee of the Faculty of Medicine of Cologne University, Cologne, Germany. The patients/participants provided their written informed consent to participate in this study.

## Author Contributions

QB, OO, GF, and BR designed the experiment. OO and BR collected the data. QB, SB, OO, NR, JD, and RF analyzed the data. QB, SB, OO, NR, JD, RF, BR, and GF wrote, revised, and approved the manuscript. All authors listed in the author list fully qualify for authorship and contributed significantly to the work.

## Conflict of Interest

The authors declare that the research was conducted in the absence of any commercial or financial relationships that could be construed as a potential conflict of interest.

## Footnotes

1 www.brainmap.org/taxonomy

2 http://dbm.neuro.uni-jena.de/cat/

3 http://www.fil.ion.ucl.ac.uk/spm/software/spm12

4 https://web.conn-toolbox.org/measures/roi-to-roi

## References

[B1] Ashburner J. (2009). Computational anatomy with the SPM software. *Magn. Reson. Imaging* 27 1163–1174. 10.1016/j.mri.2009.01.006 19249168

[B2] Balsters J. H. Laird A. R. Fox P. T. Eickhoff S. B. (2014). Bridging the gap between functional and anatomical features of cortico-cerebellar circuits using meta-analytic connectivity modeling. *Hum. Brain Mapp.* 35 3152–3169. 10.1002/hbm.22392 24142505 PMC5293143

[B3] Bakker A. Krauss G. L. Albert M. S. Speck C. L. Jones L. R. Stark C. E. (2012). reduction of hippocampal hyperactivity improves cognition in amnestic mild cognitive impairment. *Neuron* 74 467–474. 10.1016/j.neuron.2012.03.023 22578498 PMC3351697

[B4] Bassett D. S. Bullmore E. T. Meyer-Lindenberg A. Apud J. A. Weinberger D. R. Coppola R. (2009). Cognitive fitness of cost-efficient brain functional networks. *Proc. Natl. Acad. Sci. U.S.A.* 106 11747–11752. 10.1073/pnas.0903641106 19564605 PMC2703669

[B5] Bullmore E. Sporns O. (2009). Complex brain networks: graph theoretical analysis of structural and functional systems. *Nat. Rev. Neurosci.* 10 186–198. 10.1038/nrn2575 19190637

[B6] Cabeza R. (2002). Hemispheric asymmetry reduction in older adults: the HAROLD model. *Psychol. Aging* 17 85–100. 10.1037/0882-7974.17.1.85 11931290

[B7] Cabeza R. Albert M. Belleville S. Craik F. I. M. Duarte A. Grady C. L. (2018). Maintenance, reserve and compensation: the cognitive neuroscience of healthy ageing. *Nat. Rev. Neurosci.* 19 701–710. 10.1038/s41583-018-0068-2 30305711 PMC6472256

[B8] Cabeza R. Dennis N. A. (2012). Frontal lobes and aging. *Princ. Front. Lobe Funct.* 37 628–652. 10.1093/acprof:oso/9780195134971.001.0001

[B9] Cieslik E. C. Zilles K. Caspers S. Roski C. Kellermann T. S. Jakobs O. (2013). Is There “One” DLPFC in cognitive action control? evidence for heterogeneity from co-activation-based Parcellation. *Cereb. Cortex* 23 2677–2689. 10.1093/cercor/bhs256 22918987 PMC3792742

[B10] Clos M. Amunts K. Laird A. R. Fox P. T. Eickhoff S. B. Vogt O. (2013). Tackling the multifunctional nature of Broca’s region meta-analytically: co-activation-based parcellation of area 44 HHS Public access. *Neuroimage* 83 174–188. 10.1016/j.neuroimage.2013.06.041 23791915 PMC4791055

[B11] Conwell K. von Reutern B. Richter N. Kukolja J. Fink G. R. Onur O. A. (2018). Test-retest variability of resting-state networks in healthy aging and prodromal Alzheimer’s disease. *Neuroimage Clin.* 19 948–962. 10.1016/J.NICL.2018.06.016 30003032 PMC6039839

[B12] Cotelli M. Manenti R. Cappa S. F. Zanetti O. Miniussi C. (2008). Transcranial magnetic stimulation improves naming in Alzheimer disease patients at different stages of cognitive decline. *Eur. J. Neurol.* 15 1286–1292. 10.1111/j.1468-1331.2008.02202.x 19049544

[B13] Davis S. W. Dennis N. A. Daselaar S. M. Fleck M. S. Cabeza R. (2008). Qué PASA? the posterior-anterior shift in aging. *Cereb. Cortex* 18 1201–1209. 10.1093/cercor/bhm155 17925295 PMC2760260

[B14] delEtoile J. Adeli H. (2017). Graph theory and brain connectivity in Alzheimer’s disease. *Neuroscientist* 23 616–626. 10.1177/1073858417702621 28406055

[B15] Dennis M. Spiegler B. J. Juranek J. J. Bigler E. D. Snead O. C. Fletcher J. M. (2013). Age, plasticity, and homeostasis in childhood brain disorders. *Neurosci. Biobehav. Rev.* 37 2760–2773. 10.1016/j.neubiorev.2013.09.010 24096190 PMC3859812

[B16] Dimitriadis S. I. Laskaris N. A. Tsirka V. Vourkas M. Micheloyannis S. Fotopoulos S. (2010). Tracking brain dynamics via time-dependent network analysis. *J. Neurosci. Methods* 193 145–155. 10.1016/J.JNEUMETH.2010.08.027 20817039

[B17] Dubois B. Feldman H. H. Jacova C. Hampel H. Molinuevo J. L. Blennow K. (2014). Advancing research diagnostic criteria for Alzheimer’s disease: the IWG-2 criteria. *Lancet Neurol.* 13 614–629. 10.1016/S1474-4422(14)70090-0 24849862

[B18] Dubois B. Hampel H. Feldman H. H. Scheltens P. Aisen P. Andrieu S. (2016). Preclinical Alzheimer’s disease: definition, natural history, and diagnostic criteria. *Alzheimer Dement.* 12 292–323. 10.1016/j.jalz.2016.02.002 27012484 PMC6417794

[B19] Dubois J. Adolphs R. (2016). Building a science of individual differences from fMRI. *Trends Cogn. Sci.* 20 425–443. 10.1016/j.tics.2016.03.014 27138646 PMC4886721

[B20] Duits F. H. Prins N. D. Lemstra A. W. Pijnenburg Y. A. L. Bouwman F. H. Teunissen C. E. (2015). Diagnostic impact of CSF biomarkers for Alzheimer’s disease in a tertiary memory clinic. *Alzheimers Dement.* 11 523–532. 10.1016/j.jalz.2014.05.1753 25156643

[B21] Eickhoff S. B. Bzdok D. Laird A. R. Roski C. Caspers S. Zilles K. (2011). Co-activation patterns distinguish cortical modules, their connectivity and functional differentiation. *Neuroimage* 57 938–949. 10.1016/j.neuroimage.2011.05.021 21609770 PMC3129435

[B22] Enciu A. M. Nicolescu M. I. Manole C. G. Mureşanu D. F. Popescu L. M. Popescu B. O. (2011). Neuroregeneration in neurodegenerative disorders. *BMC Neurol.* 11:75. 10.1186/1471-2377-11-75 21699711 PMC3146817

[B23] Fan L. Li H. Zhuo J. Zhang Y. Wang J. Chen L. (2016). The human brainnetome atlas: a new brain atlas based on connectional architecture. *Cereb. Cortex* 26 3508–3526. 10.1093/cercor/bhw157 27230218 PMC4961028

[B24] Faul F. Erdfelder E. Lang A. G. Buchner A. (2007). G^∗^Power 3: a flexible statistical power analysis program for the social, behavioral, and biomedical sciences. *Behav. Res. Methods* 39 175–191. 10.3758/bf03193146 17695343

[B25] Fox P. T. Lancaster J. L. Laird A. R. Eickhoff S. B. (2014). Meta-analysis in human neuroimaging: computational modeling of large-scale databases. *Annu. Rev. Neurosci.* 37 409–434. 10.1146/annurev-neuro-062012-170320 25032500 PMC4782802

[B26] Fuchs E. Flügge G. (2014). Adult neuroplasticity: more than 40 years of research. *Neural Plast.* 2014:541870. 10.1155/2014/541870 24883212 PMC4026979

[B27] Gabrieli J. D. E. Ghosh S. S. Whitfield-Gabrieli S. (2015). Prediction as a humanitarian and pragmatic contribution from human cognitive neuroscience. *Neuron* 85 11–26. 10.1016/j.neuron.2014.10.047 25569345 PMC4287988

[B28] Gregory S. Long J. D. Klöppel S. Razi A. Scheller E. Minkova L. (2017). Operationalizing compensation over time in neurodegenerative disease. *Brain* 140 1158–1165. 10.1093/brain/awx022 28334888 PMC5382953

[B29] Howard M. W. Rizzuto D. S. Caplan J. B. Madsen J. R. Lisman J. Aschenbrenner-Scheibe R. (2003). Gamma oscillations correlate with working memory load in humans. *Cereb. Cortex* 13 1369–1374. 10.1093/cercor/bhg084 14615302

[B30] Huang C.-M. Polk T. A. Goh J. O. Park D. C. (2012). Both left and right posterior parietal activations contribute to compensatory processes in normal aging. *Neuropsychologia* 50 55–66. 10.1016/j.neuropsychologia.2011.10.022 22063904 PMC3355662

[B31] Huang S. Seidman L. J. Rossi S. Ahveninen J. (2013). Distinct cortical networks activated by auditory attention and working memory load. *Neuroimage* 83 1098–1108. 10.1016/j.neuroimage.2013.07.074 23921102 PMC3815975

[B32] Jack C. R. Bennett D. A. Blennow K. Carrillo M. C. Dunn B. Haeberlein S. B. (2018). NIA-AA research framework: toward a biological definition of Alzheimer’s disease. *Alzheimers Dement.* 14 535–562. 10.1016/j.jalz.2018.02.018 29653606 PMC5958625

[B33] Jack C. R. Bennett D. A. Blennow K. Carrillo M. C. Feldman H. H. Frisoni G. B. (2016). A/T/N: an unbiased descriptive classification scheme for Alzheimer disease biomarkers. *Neurology* 87 539–547. 10.1212/WNL.0000000000002923 27371494 PMC4970664

[B34] Jenkinson M. Bannister P. Brady M. Smith S. (2002). Improved optimization for the robust and accurate linear registration and motion correction of brain images. *Neuroimage* 17 825–841. 10.1006/nimg.2002.1132 12377157

[B35] Kambara T. Brown E. C. Jeong J. W. Ofen N. Nakai Y. Asano E. (2017). Spatio-temporal dynamics of working memory maintenance and scanning of verbal information. *Clin. Neurophysiol.* 128 882–891. 10.1016/j.clinph.2017.03.005 28399442 PMC5429980

[B36] Khazaee A. Ebrahimzadeh A. Babajani-Feremi A. (2016). Application of advanced machine learning methods on resting-state fMRI network for identification of mild cognitive impairment and Alzheimer’s disease. *Brain Imaging Behav.* 10 799–817. 10.1007/s11682-015-9448-7 26363784

[B37] Kinsella G. Storey E. Crawford J. R. (2007). “Executive function and its assessment,” in *Neurology and Clinical Neuroscience*, ed. A. H. V. Schapira (Amsterdam: Elsevier Inc.), 83–95. 10.1016/B978-0-323-03354-1.50011-0

[B38] Kirschen M. P. Chen S. H. A. Desmond J. E. (2010). Modality specific cerebro-cerebellar activations in verbal working memory: an fMRI study. *Behav. Neurol.* 23 51–63. 10.3233/BEN-2010-0266 20714061 PMC2944406

[B39] Laird A. R. Eickhoff S. B. Kurth F. Fox P. M. Uecker A. M. Turner J. A. (2009). ALE meta-analysis workflows via the brainmap database: progress towards a probabilistic functional brain atlas. *Front. Neuroinform.* 3:23. 10.3389/neuro.11.023.2009 19636392 PMC2715269

[B40] Linden D. E. J. (2012). The challenges and promise of neuroimaging in psychiatry. *Neuron* 73 8–22. 10.1016/j.neuron.2011.12.014 22243743

[B41] Logan J. M. Sanders A. L. Snyder A. Z. Morris J. C. Buckner R. L. (2002). Under-recruitment and nonselective recruitment: dissociable neural mechanisms associated with aging. *Neuron* 33 827–840. 10.1016/S0896-6273(02)00612-8 11879658

[B42] Lövdén M. Bäckman L. Lindenberger U. Schaefer S. Schmiedek F. (2010). A theoretical framework for the study of adult cognitive plasticity. *Psychol. Bull.* 136 659–676. 10.1037/a0020080 20565172

[B43] Lux S. Helmstaedter C. Elger C. E. (1999). Normierungsstudie zum Verbalen Lern- und Merkfähigkeitstest (VLMT). *Diagnostica* 45 205–211. 10.1026//0012-1924.45.4.205

[B44] McCarthy P. Benuskova L. Franz E. A. (2014). The age-related posterior-anterior shift as revealed by voxelwise analysis of functional brain networks. *Front. Aging Neurosci.* 6:301. 10.3389/fnagi.2014.00301 25426065 PMC4224138

[B45] Mechelli A. Price C. J. Friston K. J. Ashburner J. (2005). Voxel-based morphometry of the human brain: methods and applications. *Curr. Med. Imag.* 1:726.

[B46] Meltzer J. A. Zaveri H. P. Goncharova I. I. Distasio M. M. Papademetris X. Spencer S. S. (2008). Effects of working memory load on oscillatory power in human intracranial EEG. *Cereb. Cortex* 18 1843–1855. 10.1093/cercor/bhm213 18056698 PMC2474453

[B47] Narayanan N. S. Prabhakaran V. Bunge S. A. Christoff K. Fine E. M. Gabrieli J. D. E. (2005). The role of the prefrontal cortex in the maintenance of verbal working memory: an event-related fMRI analysis. *Neuropsychology* 19 223–232. 10.1037/0894-4105.19.2.223 15769206

[B48] Noy N. Bickel S. Zion-Golumbic E. Harel M. Golan T. Davidesco I. (2015). Intracranial recordings reveal transient response dynamics during information maintenance in human cerebral cortex. *Hum. Brain Mapp.* 36 3988–4003. 10.1002/hbm.22892 26147431 PMC6869725

[B49] Oldfield R. C. (1971). The assessment and analysis of handedness: the Edinburgh inventory. *Neuropsychologia* 9 97–113. 10.1016/0028-3932(71)90067-4 5146491

[B50] Petersen R. C. Doody R. Kurz A. Mohs R. C. Morris J. C. Rabins P. V. (2001). Current concepts in mild cognitive impairment. *Arch. Neurol.* 58 1985–1992. 10.1001/archneur.58.12.1985 11735772

[B51] Piefke M. Onur ÖA. Fink G. R. (2012). Aging-related changes of neural mechanisms underlying visual-spatial working memory. *Neurobiol. Aging* 33 1284–1297. 10.1016/j.neurobiolaging.2010.10.014 21130531

[B52] R Core Team (2013). *A Language and Environment for Statistical Computing.* Vienna: R Foundation for Statistical Computing.

[B53] Reuter-Lorenz P. A. Cappell K. A. (2008). Neurocognitive aging and the compensation hypothesis. *Curr. Direc. Phychol. Sci.* 17 177–182. 10.1111/j.1467-8721.2008.00570.x

[B54] Rodewald K. Bartolovic M. Debelak R. Aschenbrenner S. Weisbrod M. Roesch-Ely D. (2012). Eine normierungsstudie eines modifizierten trail making tests im deutschsprachigen raum. *Zeitschrift Neuropsychol.* 23 37–48. 10.1024/1016-264X/a000060

[B55] Schmahmann J. D. (2010). The role of the cerebellum in cognition and emotion: personal reflections since 1982 on the dysmetria of thought hypothesis, and its historical evolution from theory to therapy. *Neuropsychol. Rev.* 20 236–260. 10.1007/s11065-010-9142-x 20821056

[B56] Solé-Padullés C. Bartrés-Faz D. Junqué C. Clemente I. C. Molinuevo J. L. Bargalló N. (2006). Repetitive transcranial magnetic stimulation effects on brain function and cognition among elders with memory dysfunction. a randomized sham-controlled study. *Cereb. Cortex* 16 1487–1493. 10.1093/cercor/bhj083 16339086

[B57] Wang J. (2010). Graph-based network analysis of resting-state functional MRI. *Front. Syst. Neurosci.* 4:16. 10.3389/fnsys.2010.00016 20589099 PMC2893007

[B58] Whitfield-Gabrieli S. Nieto-Castanon A. (2012). *Conn*: a functional connectivity toolbox for correlated and anticorrelated brain networks. *Brain Connect.* 2 125–141. 10.1089/brain.2012.0073 22642651

[B59] Wickham H. (2016). *ggplot2*, 2nd Edn. New York City, NY: Springer International Publishing. 10.1007/978-3-319-24277-4

[B60] Yan C.-G. Cheung B. Kelly C. Colcombe S. Craddock R. C. Di Martino A. (2013). A comprehensive assessment of regional variation in the impact of head micromovements on functional connectomics. *Neuroimage* 76 183–201. 10.1016/j.neuroimage.2013.03.004 23499792 PMC3896129

[B61] Yao Z. Zhang Y. Lin L. Zhou Y. Xu C. Jiang T. (2010). Abnormal cortical networks in mild cognitive impairment and Alzheimer’s disease. *PLoS Comput. Biol.* 6:e1001006. 10.1371/journal.pcbi.1001006 21124954 PMC2987916

[B62] Yarkoni T. Westfall J. (2017). Choosing prediction over explanation in psychology: lessons from machine learning. *Perspect. Psychol. Sci.* 12 1100–1122. 10.1177/1745691617693393 28841086 PMC6603289

[B63] Zhao Q. Guo Q. Liang X. Chen M. Zhou Y. Ding D. (2015). Auditory verbal learning test is superior to rey-osterrieth complex figure memory for predicting mild cognitive impairment to Alzheimer’s disease. *Curr. Alzheimer Res.* 12 520–526. 10.2174/1567205012666150530202729 26027810

